# An affinity-structure database of helix-turn-helix: DNA complexes with a universal coordinate system

**DOI:** 10.1186/s12859-015-0819-2

**Published:** 2015-11-19

**Authors:** Mohammed AlQuraishi, Shengdong Tang, Xide Xia

**Affiliations:** Department of Systems Biology, Harvard Medical School, Boston, MA 02115 USA; HMS Laboratory of Systems Pharmacology, Harvard Medical School, 200 Longwood Avenue, Boston, MA 02115 USA

**Keywords:** Protein-DNA, Database, Helix-turn-helix, Transcription factors, Structure, PWM

## Abstract

**Background:**

Molecular interactions between proteins and DNA molecules underlie many cellular processes, including transcriptional regulation, chromosome replication, and nucleosome positioning. Computational analyses of protein-DNA interactions rely on experimental data characterizing known protein-DNA interactions structurally and biochemically. While many databases exist that contain either structural or biochemical data, few integrate these two data sources in a unified fashion. Such integration is becoming increasingly critical with the rapid growth of structural and biochemical data, and the emergence of algorithms that rely on the synthesis of multiple data types to derive computational models of molecular interactions.

**Description:**

We have developed an integrated affinity-structure database in which the experimental and quantitative DNA binding affinities of helix-turn-helix proteins are mapped onto the crystal structures of the corresponding protein-DNA complexes. This database provides access to: (i) protein-DNA structures, (ii) quantitative summaries of protein-DNA binding affinities using position weight matrices, and (iii) raw experimental data of protein-DNA binding instances. Critically, this database establishes a correspondence between experimental structural data and quantitative binding affinity data at the single basepair level. Furthermore, we present a novel alignment algorithm that structurally aligns the protein-DNA complexes in the database and creates a unified residue-level coordinate system for comparing the physico-chemical environments at the interface between complexes. Using this unified coordinate system, we compute the statistics of atomic interactions at the protein-DNA interface of helix-turn-helix proteins. We provide an interactive website for visualization, querying, and analyzing this database, and a downloadable version to facilitate programmatic analysis.

**Conclusions:**

This database will facilitate the analysis of protein-DNA interactions and the development of programmatic computational methods that capitalize on integration of structural and biochemical datasets. The database can be accessed at http://ProteinDNA.hms.harvard.edu.

## Background

Protein-DNA interactions are among the most fundamental molecular interactions in the cell, underlying transcriptional regulation, chromosome replication, repair, and segregation, nucleosome positioning, plus many other processes. Owing to their central role in biology, protein-DNA interactions have been extensively analyzed and modeled using a variety of computational approaches. These approaches have traditionally been either sequence-based or structure-based. Sequence-based methods model the DNA-binding affinity of a protein using its known DNA binding sites and range in complexity from simple models such as consensus sequences and position-weight matrices (PWMs) to complex models like Variable-Order Bayesian Networks and Feature Motif Models [1–6]. Data from experimental methods such as DNA footprinting [7, 8], SELEX [9], ChIP-seq [10, 11], and microarrays [12] are used to derive such models. In contrast, structure-based methods predict the DNA binding affinity of a protein from its molecular structure—obtained either computationally or by experimental methods such as X-ray crystallography and NMR—and its predicted orientation vis-a-vis different DNA sequences, by employing an energy function to compute the protein-DNA binding energy [13–17]. The energy functions that have been used in structure-based methods are derived either from theory or from statistics of interatomic contacts in crystallized protein-DNA structures.

Many databases have been developed that address the particular needs of the sequence- and structure-based approaches. On the sequence side, DNA-binding site databases such as TRANSFAC [18], JASPAR [19], and others provide accessibility to raw binding site data and simple models of protein-DNA binding affinity like PWMs. Specialty databases that include quantitative binding-affinity data also exist, such as ProNIT [20], UniPROBE [12]. On the structure-side, databases like the Protein Data Bank (PDB) [21] provide general access to protein structures, and specialty databases such as NPIDB [22] and BIPA [23] provide culled resources containing only protein-DNA complexes.

While these databases have proven satisfactory for addressing the needs of computational methods that fall squarely into one category or another, the development of algorithmic techniques that utilize both sequence and structural data necessitates an integrative database that couples protein-DNA structural complexes with their binding affinity. In particular, merely curating structural and binding affinity is insufficient. For algorithms to exploit the association between structural properties and quantitative binding affinity, a correspondence must be established between every DNA basepair position in a protein-DNA structural complex and the protein’s experimentally-determined binding affinity for different nucleotides at that position. In this way, supervised machine learning algorithms can use structural properties as inputs and binding affinity as output to learn models that can predict protein-DNA interactions. To our knowledge none of the databases currently combining structural and binding affinity data, including TFinDit [24] and 3d-footprint [25], provide such a correspondence.

**Fig. 1:**
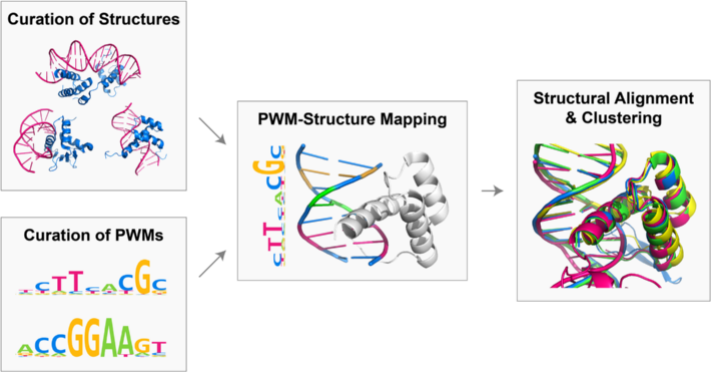
Database creation steps. Protein-DNA structures and position weight matrices (PWMs) were first curated from existing databases and the primary literature. Newly developed structural alignments algorithms were then used to establish a correspondence between structures and PWMs, by associating a probability distribution with every residue in every protein-DNA structural complex. Finally the resulting structures were clustered and aligned to establish a universal coordinate system across all helix-turn-helix domains

We report the development of a database of protein-DNA structural complexes that provides this correspondence. We previously used this database to derive a new class of machine-learning-based protein-DNA energy potentials that utilize structural data and binding affinities [26, 27]. Our database contains novel features that make it suitable for general use in the analysis of the relationship between sequence and structure. First, the atomic structures of 63 protein-DNA complexes are combined with probabilistic information regarding the likelihood of binding of every basepair in the structure. These probabilities were specifically derived for this database, by analysis of many primary sources and secondary databases. We determined a probability distribution for the likelihood of binding different nucleotides at every DNA basepair position in the set of protein-DNA complexes in the database. Second, we developed a novel structural alignment and clustering algorithm that performs a structural superpositioning of all the protein-DNA complexes in the database. This enabled us to derive a single coordinate system to index all DNA basepair positions and all amino-acid residues in the binding interface of the protein-DNA complexes to facilitate analysis and comparison of the physico-chemical environments that surround the bases and residues involved in protein-DNA binding. In deriving this unified coordinate system we focused on one protein family with a single DNA-binding modality. We chose the helix-turn-helix (HTH) [28] family as it is the most widely distributed family of DNA-binding proteins, occurring in all biological kingdoms and with a large number of crystallized structures. Also, virtually all bacterial transcription factors are HTH proteins as are about one-fourth of human transcription factors [29]. Finally, to facilitate their use in automated programmatic analysis, all the protein-DNA complexes in the database have been processed to standardize their chain ordering and connectivity, and to remove any pathologies. We provide this database in downloadable form and in an interactive website that can be used to browse and visualize the protein-DNA binding interface of all complexes. Figure 1 contains an overview of the database assembly process.

By integrating binding information from dozens of sources, presenting a unified probabilistic formulation to describe the DNA-binding affinity of proteins, mapped directly onto the atomic structures of aligned protein-DNA complexes, and creating a unified coordinate system to analyze and compare these structures, we have constructed a database that will be a valuable and unique resource for researchers.

## Construction and content

### Curation of protein-DNA structures

**Fig. 2:**
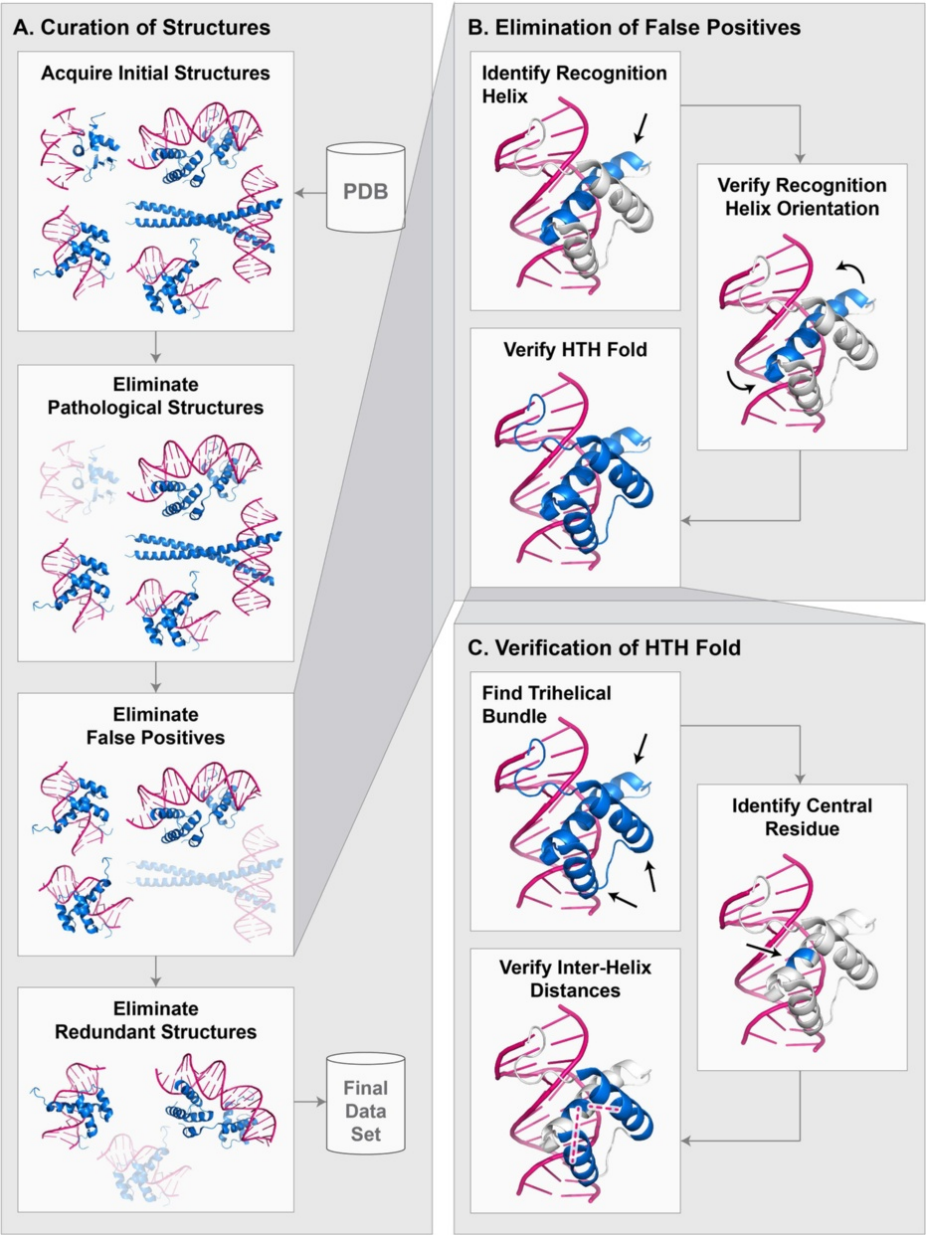
Curation of protein-DNA structures. **a** End-to-end process for curating protein-DNA structures. **b** Eliminating false positives. **c** Verifying that a protein contains an HTH domain

To curate protein-DNA atomic structures, we developed a largely automated pipeline beginning with the initial data acquisition step that retrieves all HTH-DNA complexes from the PDB, followed by several elimination steps that remove inappropriate and redundant structures, and finally a processing step that prepares the structures for use in programmatic analysis (Fig. 2a). The initial operation in the pipeline is a systematic search for all atomic structures of HTH-DNA complexes in the PDB. Since HTH domains are found in many distinct subfamilies, with inconsistent naming conventions across different classification schemes, we developed our own search criteria. Multiple searches were performed to obtain all the sought structures in the PDB. Table 1 shows the settings common to all searches. In addition to the common search settings, each query targeted a particular structural family. Table 2 lists the structural families that were used as targets. We found that the combined criteria minimize false negative and false positives, i.e. the searches missed very few, if any, HTHs and retrieved few non-HTHs. The structures retrieved during this step were then fed into a sequence of elimination steps that removed anomalous structures based on several criteria, including the presence of structural pathologies, false positives, and redundancies (described below).

**Table 1:** PDB search settings for all HTH-DNA retrieval settings. Indented rows indicate sub-fields

Search field	Setting
Macromolecule type	
Contains protein	Yes
Contains DNA	Yes
Contains RNA	No
Contains DNA/RNA hybrid	No
Methods	
Experimental method	X-RAY

**Table 2:** Structural families used as target queries to retrieve HTH-DNA structures. Indented rows indicate sub-fields, and multiple columns under “Setting” indicate a hierarchical choice

Search field	Setting			
Structure features				
SCOP	All α	DNA/RNA-binding 3-helical bundle (core: 3-helices; bundle, closed or partly opened, right-handed twist; up-and down)		
SCOP	All α	lambda repressor-like DNA-binding domains		
SCOP	All α	Cyclin-like	TFIIB	
CATH	Mainly α	Orthogonal Bundle	Arc Repressor Mutant, subunit A	1.10.10.10
CATH	Mainly α	Orthogonal Bundle	Arc Repressor Mutant, subunit A	1.10.10.60
CATH	Mainly α	Orthogonal Bundle	434 Repressor (Amino-terminal Domain)	1.10.260.40
CATH	α/β	2-Layer Sandwich	CRO Repressor	3.30.240.10
CATH	Mainly α	Orthogonal Bundle	Arc Repressor Mutant, subunit A	1.10.10.400
CATH	Mainly α	Orthogonal Bundle	Factor For Inversion Stimulation; Chain: A	1.10.1680.10
CATH	Mainly α	Orthogonal Bundle	Chromosomal Replication Initiator Protein DnaA; Chain: A	1.10.1750.10
CATH	Mainly α	Orthogonal Bundle	Trp Operon Repressor; Chain A	1.10.1270.10
CATH	Mainly α	Orthogonal Bundle	Arc Repressor Mutant, subunit A	1.10.10.200
CATH	Mainly α	Orthogonal Bundle	Arc Repressor Mutant, subunit A	1.10.10.500
CATH	Mainly α	Orthogonal Bundle	Tetracycline Repressor; domain 2	1.10.357.10
CATH	Mainly α	Orthogonal Bundle	Putative cytoplasmic protein	1.10.3100.10
CATH	Mainly α	Orthogonal Bundle	Arc Repressor Mutant, subunit A	1.10.10.560
CATH	Mainly α	Orthogonal Bundle	Arc Repressor Mutant, subunit A	1.10.10.570
CATH	Mainly α	Orthogonal Bundle	Arc Repressor Mutant, subunit A	1.10.10.580
CATH	Mainly α	Orthogonal Bundle	Apoptosis Regulator Bcl-x	1.10.437.10
CATH	Mainly α	Orthogonal Bundle	Arc Repressor Mutant, subunit A	1.10.10.250

#### Elimination of pathological structures

Complexes with three types of structural pathologies were eliminated: (i) the DNA is single-stranded instead of double-stranded, (ii) the complex contains missing backbone atoms, specifically C_α_ atoms for proteins and C_1’_, C_2’_, C_3’_, C_4’_, and C_5’_ atoms for DNA, and (iii) the protein contains non-standard amino acid residues. The elimination of such pathologies streamlines the analysis and insures that only atomically accurate structures are considered.

#### Elimination of false positive structures

**Fig. 3:**
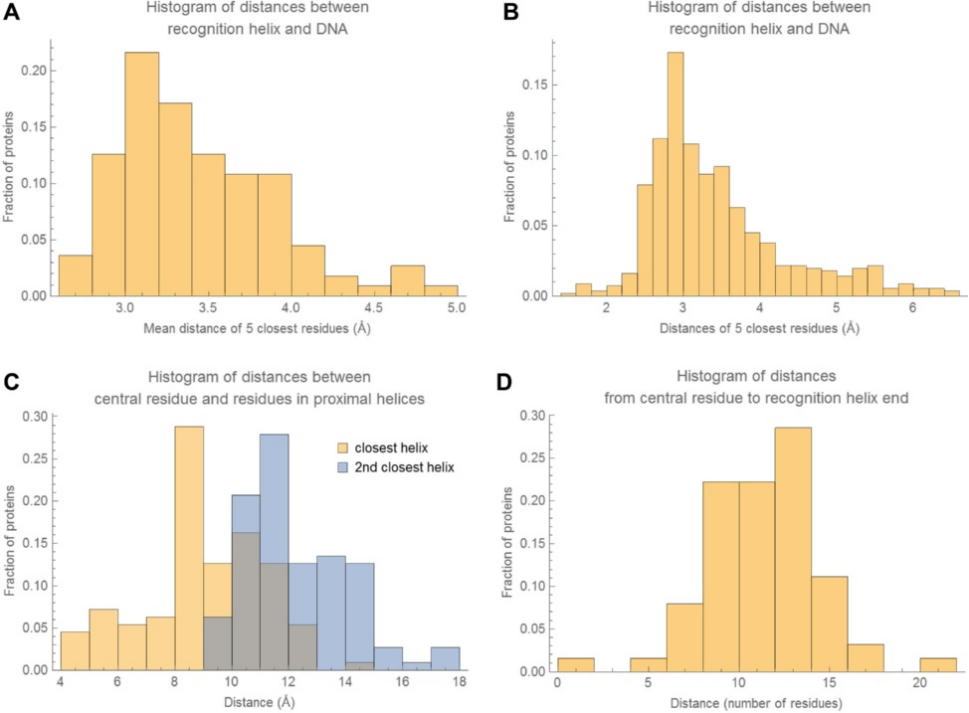
Statistical distribution of HTH structural properties. All histograms show distribution over HTH domains in our database. **a** Histogram of mean distances of 5 closest residues of recognition helix to DNA. **b** Histogram of all distances of five closest residues of recognition helix to DNA. **c** Histograms of distances between central residue of recognition helix (*see text for description*) and residues in closest and 2nd closest α-helices. **d** Histogram of residue distances along protein chain from central residue to recognition helix end

Our initial search criteria retrieved a number of domains that we identified as false positives (i.e. non-HTH domains) through manual inspection. Based on the true HTHs, we developed several heuristics that, when used in concert, eliminated the vast majority of non-HTH domains (Fig. 2b). Some of these heuristics rely on numerical parameters, such as the separation between helices, which we derived based on the statistics of structural properties of HTH domains (Fig. 3). Specifically, the algorithm first finds a putative α-helix that we consider to be a candidate recognition helix. Our criterion for candidacy is that the mean distance between the closest five residues (contiguous) of the α-helix to the DNA molecule is less than 5 Å. This criterion insures that the α-helix is sufficiently close to make contact with the DNA molecule. Any number of residues can be used for computing the mean distance, but we chose five residues as that is close to the smallest recognition helix present in our database. Figure 3a depicts the distribution of these mean distances to the DNA molecule for true HTH domains. Based on this distribution, we chose 5 Å as the cutoff. We define the distance between an α-helix residue and a DNA molecule to be the shortest pairwise distance over all atoms in the residue and all atoms in the DNA molecule. Once an α-helix is identified as a putative recognition helix, the second step is to insure that its orientation relative to the DNA is correct. We found that an effective heuristic for insuring correct orientation is to require that each of the five closest residues are individually within a certain distance cutoff of the DNA. Figure 3b depicts the distributions of these distances for true HTH domains. Based on this distribution, we chose 6.5 Å as the cutoff. Distance is computed in the same way as in the first step. Finally, a third step is taken to insure that the entire domain is in fact an HTH motif, by detecting the core tri-helical bundle that is representative of all HTHs [28]. The distance calculations in this step are more complex, owing to the variability of α-helix lengths between HTH domains. An overview of the process is shown in Fig. 2c. First, at least three distinct α-helices must be detected. Second, a “central residue” is identified within the putative recognition helix that represents the centermost point of contact with the DNA molecule. This is done by computing the major axis of the DNA molecule [30] and then computing the distance between this axis and the C_α_ atom of each residue in the putative recognition helix. The residue closest to the major axis of the DNA molecule is considered the central residue. Once the central residue is identified, the pairwise distances between the C_α_ atom of the central residue and the C_α_ atoms of all the residues in the other α-helices are computed. Figure 3c depicts the distributions of these distances with respect to the closest and second closest α-helices for true HTH domains. Based on these distributions, we classify a tri-helical structure as an HTH if the central residue is within 18 Å of at least one residue in two distinct α-helices. While individually the described heuristics do not eliminate all non-HTH structures, we found that in concert they eliminate the vast majority, making it feasible to remove the remaining false positives manually.

#### Elimination of redundant structures

**Fig. 4:**
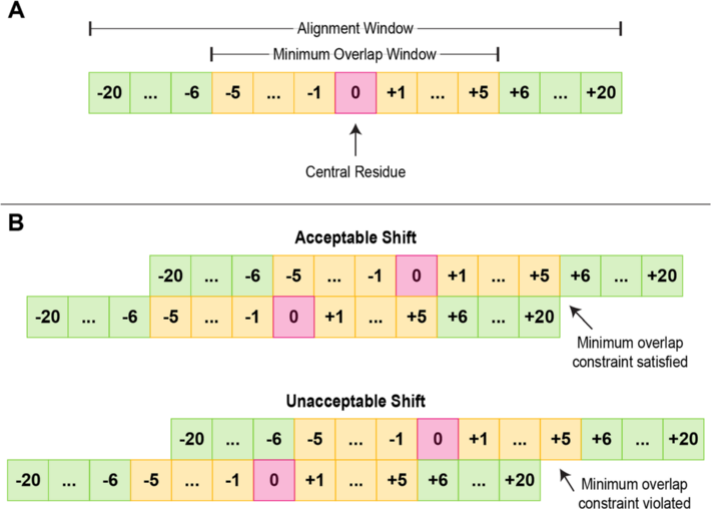
Criteria for comparing two recognition helices. **a** Schematic depiction of a recognition helix, with each residue position represented by a numbered square whose number refers to the residue position. The central residue is the 0th position. A 41-residue window centered on the central residue is used as the basis for comparing two HTH domains. An 11-residue window flanking the central residue defines the minimum region of overlap for a recognition helix. **b** Examples illustrating allowable (*top*) and unallowable (*bottom*) shifts between two helices. Only allowable shifts are used when computing the distance between two helices

**Fig. 5:**
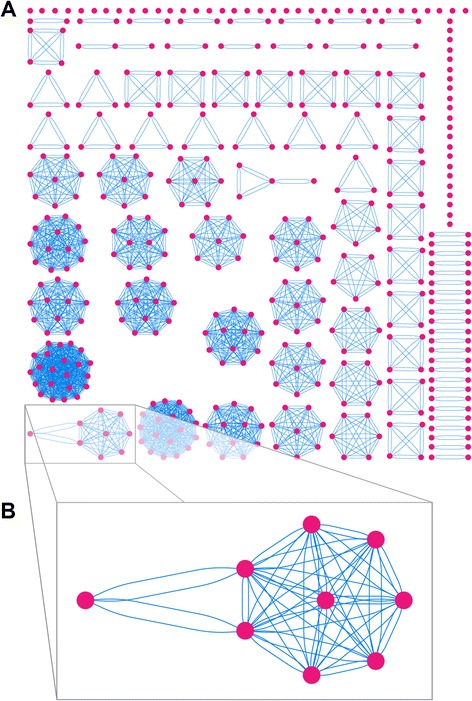
Visualizing distances between recognition helices as a graph. **a** Nodes (*pink circles*) represent individual recognition helices. Edges (*blue lines*) are formed between two nodes if their respective recognition helices have a sequence distance of zero. Disconnected nodes (*pink circles with no edges*) are unique HTH domains. **b** Some subgraphs are not fully connected, i.e. not every node is connected to every other node. In such cases the central-most node with the highest resolution is chosen

As the primary purpose of this database is to enable machine learning applications, we removed redundant complexes to prevent algorithms trained on this database from overfitting on overrepresented structures. We consider complexes redundant if they have the same amino acid sequence in the region of the recognition α-helix. We chose this criterion due to the dominant role that recognition α-helices play in effecting the sequence specificity of HTH proteins, and the fact that HTHs with otherwise highly similar sequences may still exhibit differential DNA binding properties [31, 32]. To identify redundant structures, the amino acid sequence of the recognition helix of every HTH domain was extracted. The recognition sequence is centered at the central residue of the recognition helices, and extended by 20 amino acids on both sides of the central residue, for a total of 41 amino acids (Fig. 4a). We chose this criterion because the recognition helices observed in our data set extended in length up to 20 amino acids on either side of the central residue, and we sought a consistent criteria to apply to all structures (Fig. 3d). The pairwise distance between every pair of HTH domains is computed using the resulting sequences. Since two recognition helices may be shifted with respect to one another, we computed the pairwise sequence distance by considering all possible shifts between two recognition helices, and the number of mismatched residues for every possible shift. The shift that gave the smallest number of mismatched residues was selected, and the number of mismatched residues returned as the distance. To ensure that the shifts are small, we require that an 11-residue window flanking the central residue is the minimum amount of overlap present between two recognition helices (Fig. 4b). We chose this criterion as it corresponds to the shortest recognition helices observed in our database. Using the resulting sequences, we formed a graph where each node represents a recognition helix and two nodes are connected by an edge if the sequence distance (Hamming distance) between their respective recognition helices is 0 (Fig. 5a). Disconnected nodes, i.e. nodes that have no edges, represent unique HTH domains by our definition and are retained in the data set. Fully connected subgraphs, i.e. those in which every node is connected to every other node, represent a subset of HTHs that are identical. From each such subgraph, only the highest resolution crystal structure is retained. In some subgraphs, owing to the distance metric used, some nodes are connected to all other nodes (Fig. 5b). In such instances the structure corresponding to the node with the largest number of edges (i.e. the most central node) is used. If there are several such structures, then the one with the highest resolution is used.

#### Processing step

After the final set of protein-DNA complexes was selected, we used a sequence of processing steps to generate a uniform set of PDB files that can be readily used in computational analysis. First, we processed all dsDNA molecules to conform to a standardized format in which the two strands of DNA are treated as separate chains, the chains are ordered in a 5′ to 3′ orientation, all overhangs are removed, and the basepairs aligned so that they are physically matched. Since many structures in the PDB do not conform to this standard, we developed scripts to reformat all PDB files in the database accordingly. Second, we extracted protein chains with multiple HTH domains and single HTH domains that span multiple chains, and formatted these protein chains so that each individual HTH domain is spanned by a single chain in an individual PDB file, along with its cognate DNA molecule. Finally, we processed the final set of PDB files with the PDB2PQR [33, 34] utility to carry out the protonation and dewatering steps. PDB2PQR is run with default settings using the AMBER molecular mechanics force field [35].

### Curation of PWMs and structure mapping

We curated experimentally-determined DNA binding sites for each of the protein-DNA structural complexes in the database. The set of binding sites was compiled from several data repositories such as TRANSFAC along with primary sources [9, 12, 16, 18–20, 36–74]. All the DNA binding sites in the database are based on experimentally assays. In some instances, the same experiment was reported in two or more of the data repositories we used. When possible (e.g. by checking the original PMID reference from which experiment is derived), we removed such redundant entries to insure that each binding site entry in the database corresponds to a unique experiment. Multiple distinct experiments reporting on the same binding site were retained however. The experimental assay and, when available, quality ratings of binding sites included in the original data repository are cited in the database (e.g. TRANSFAC quality scores). Using these DNA binding sites we generated an experimentally-derived PWM for each of the protein-DNA complexes in the database. The PWMs were derived by setting the probability of every nucleotide at every position to its empirically-observed relative frequency in the database. For positions for which we did not have any data, we used a uniform distribution over the four nucleotides as a non-informative prior. We also used Laplace smoothing to mitigate errors due to small sample size. Since the orientation and length of the binding sites varied between and within data sources, manual and automated alignment methods were used in constructing the PWMs, which were then mapped onto the protein-DNA structures so that for every basepair position in every protein-DNA complex, we maintain a probability distribution over all four possible nucleotides.

### Structural alignment

We structurally superimposed all protein-DNA complexes in the database, to establish an alignment between DNA basepairs in one complex to another, and between the amino acid residues of the recognition helices of the proteins. While in general this is not possible for any two arbitrary DNA-binding proteins, proteins within the same structural family typically exhibit a conserved modality for binding. In particular, the HTH family of proteins uses a highly conserved mode of docking into the major groove of DNA [75–77]. This suggested that it would be possible to align all HTH-DNA complexes in the database such that the DNA molecules and recognition helices are superimposed. We developed a novel structural alignment algorithm for this purpose, and used it for a pairwise alignment of all complexes in the database.

We formulated the structural alignment problem as the following optimization problem. Let RMSD_DNA_ be the root mean square deviation (RMSD) between the backbone carbon atoms of two DNA molecules, and RMSD_HTH_ be the RMSD between the C_α_ atoms of two recognition helices. Then we defined the optimal alignment as the one (over all possible alignments) that minimizes RMSD_HTH_ subject to RMSD_DNA_ < δ. The parameter δ was set to 2 Å. We solved this problem using the following four-step algorithm.

#### Canonical matching regions

**Fig. 6:**
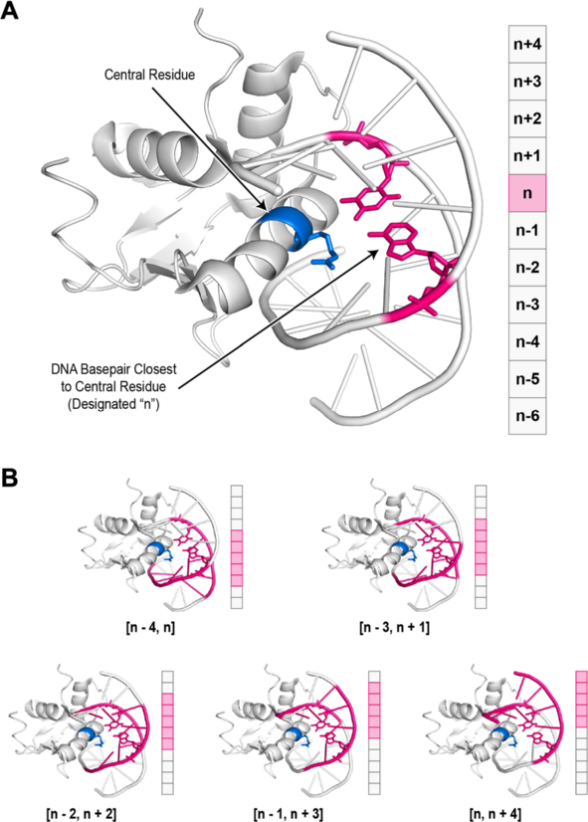
Canonical matching regions. Canonical matching regions are contiguous stretches of DNA in close proximity to the recognition helix. **a** To establish a canonical matching region, the DNA basepair position closest to the central residue of the recognition helix is identified (designated “n”). A schematic representation of all residue positions is shown on the right. **b** Multiple canonical matching regions, spanning five basepairs and constrained to include the nth DNA basepair position, are shown in pink highlights. The range of positions spanned by each region is shown in brackets under each respective complex, and depicted schematically on the right

The first step is to generate “canonical matching regions” for each HTH-DNA complex in the database. We define a canonical matching region to be a contiguous stretch of five basepairs of the DNA molecule that is in close proximity to the recognition helix of the HTH domain. Depending on the proximity criteria, many such regions exist. Our motivation for defining these regions is to use them as a basis for aligning the DNA molecules, in lieu of using the entire structure. We have found through analyses of HTH-DNA complexes that DNA molecules exhibit significant variation in their bending far away from the region of binding, i.e. the recognition helix, but are highly uniform in shape closest to the recognition helix. Therefore by basing the alignment on the region closest to binding, we increase the robustness of the resulting alignments. To define the canonical matching regions for a given HTH-DNA complex, we begin by identifying the central residue of the recognition helix, as described earlier. Once the central residue has been identified, we find the closest DNA basepair in the docked DNA molecule (Fig. 6a). Distance is defined as that between the C_α_ atom of the central residue and the closest (of the two) C_1’_ atoms of the DNA basepair. Designating the position of the closest DNA basepair by n, five distinct canonical matching regions are defined, each spanning 5 bp stretches, starting with position n − 4 and ending with position n (Fig. 6b).

#### DNA-based alignment

**Fig. 7:**
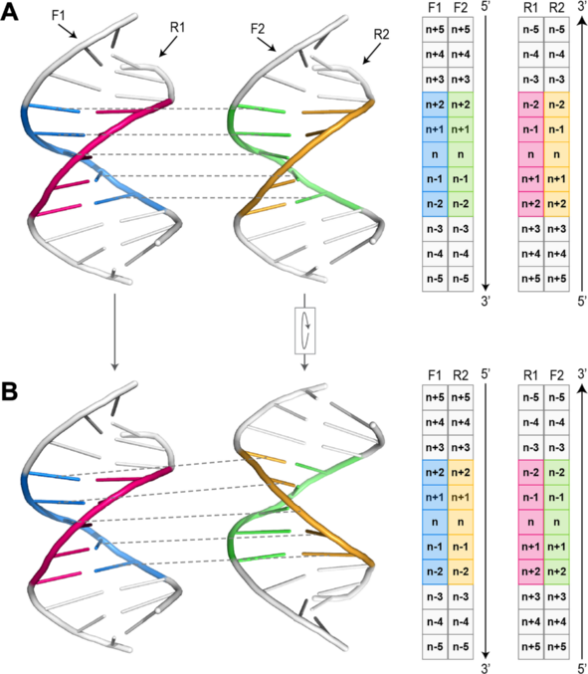
Mirror symmetry of DNA molecules. For any two canonical matching regions in different DNA molecules there are two possible alignments due to the mirror symmetry of DNA. **a** The strands F1 and R1 are mapped to F2 and R2, respectively (mapping between F1 and F2 is depicted by dashed lines). **b** The F2/R2 DNA molecule is rotated 180° around the horizontal axis, which results in strands F1 and R1 being mapped to strands R2 and F2, respectively. Schematic representations of the mappings between strands are shown on the right. Note that both alignments preserve the 5′ → 3′ directionality of DNA, i.e. alignments in which the mapped regions run in opposite directions are not allowed

**Fig. 8:**
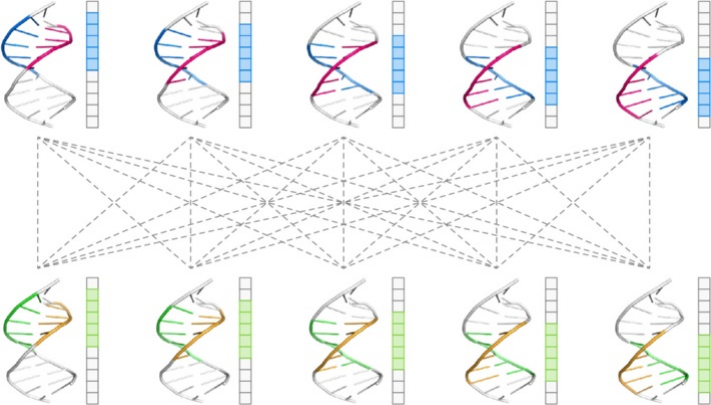
Pairwise alignments based on canonical matching regions. The two rows of DNA structures in this figure represent two HTH-DNA complexes, for which there are 50 possible pairwise alignments. Each complex has five canonical matching regions (depicted schematically to the right of every complex), which can be matched with any of the five canonical matching regions of the other HTH-DNA complex, yielding 25 possible pairings (*represented by dashed lines*). Since each pairing can yield two distinct alignments (Fig. 6), there are 50 possible alignments

**Fig. 9:**
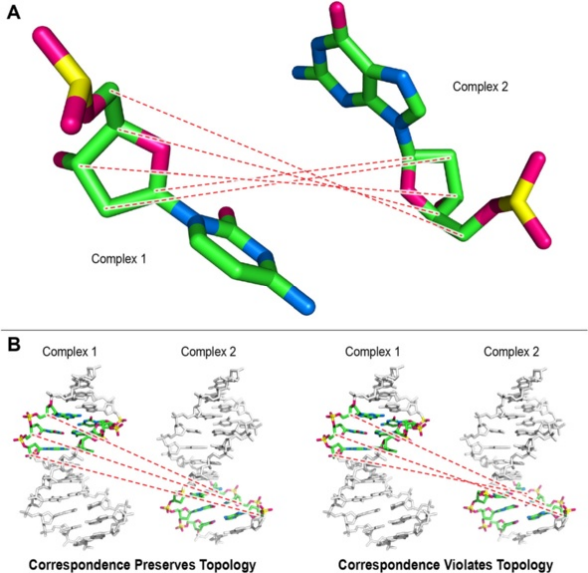
Constraints for ICP correspondence. **a** Atom types are respected when performing ICP, so that a C1’ atom in one complex always corresponds to a C1’ atom in another complex. An example of a correspondence between the backbone atoms of two nucleotides in two complexes is shown. **b** Topology is preserved when performing ICP, so that the relative order of atoms (as defined by the DNA sequence) is the same between corresponded atoms. Left correspondence shows a correct (topology-preserving) example, while the right correspondence shows an incorrect (topology-violating) example

Based on the canonical matching regions defined in the first step, all possible pairwise alignments were performed for each pair of HTH-DNA complexes. Since there are five canonical matching regions in each HTH-DNA complex, and since any two such regions can be aligned in two orientations based on the mirror symmetry of the DNA molecule (Fig. 7), there are a total of 5 × 5 × 2 = 50 possible pairwise alignments for each pair of HTH-DNA complexes (Fig. 8). All such alignments were performed, and their RMSD computed, then the alignments scoring an RMSD of more than δ were eliminated from further consideration, and the remainder were used as putative initial alignments in the next step. To carry out the pairwise alignments between two HTH-DNA complexes, we use the iterative closest points (ICP) algorithm [78]. The ICP algorithm finds an affine transformation (translation + rotation) that brings two point clouds in closest correspondence. We apply the ICP algorithm on the point clouds represented by the backbone atoms of the DNA molecules, specifically the C_1’_, C_2’_, C_3’_, C_4’_, and C_5’_ atoms. Only the DNA basepairs in the canonical matching regions are considered for this purpose. When running ICP, constraints are enforced to insure that only atoms of the same type are put in correspondence, i.e. a C_1’_ atom from one complex must map to a C_1’_ atom in the other complex (Fig. 9a). Furthermore, the topologies of the molecules have to be preserved, so that atoms from sequential basepairs in one DNA molecule map to atoms in sequential basepairs in the other DNA molecule (Fig. 9b). These constraints ensure that only physically realizable alignments are performed, while at the same time returning all 50 possible pairwise alignments for each pair of HTH-DNA complexes. Finally the RMSD score resulting from a pairwise alignment of the two canonical matching regions is computed based on the backbone atoms of the DNA basepairs in the alignment.

#### Recognition helix-based alignment

**Fig. 10:**
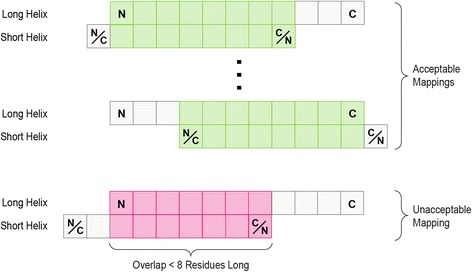
Mappings between recognition helices. All possible mappings of two recognition helices are shown schematically, where each recognition helix is represented by a series of squares. The longer recognition helix is held fixed, and is always oriented from the N- to the C-terminus. The shorter recognition helix is allowed to slide along the longer helix, as long as a minimum of 8 residues are overlap-ping between the two helices. The orientation of the shorter helix can also be flipped. Acceptable mappings are shown in green, and an unacceptable mapping is shown in pink

The 50 alignments obtained for each pair of HTH-DNA complexes serve as a set of putative alignments, from which one final alignment is selected for each pair of HTH-DNA complexes, based on the recognition helices of the HTH domains in the complexes being aligned. To select the final alignment, all pairwise alignments for a given pair of complexes are considered individually. For each alignment, the RMSD between the two recognition helices in the corresponding complexes is computed. This RMSD is calculated based on the distances between the C_α_ atoms of the amino acid residues of the two recognition helices. To compute these distances, the residues of one recognition helix must be mapped onto the residues of the other recognition helix, so that the distances between their respective C_α_ atoms can be calculated. Since a residue-by-residue mapping between the two recognition helices is not known *a priori*, we considered all such mappings, and the one yielding the lowest RMSD was selected. For each pair of recognition helices, the mappings considered include all possible shifts of one recognition helix with respect to the other one, assuming one of the recognition helices is shorter than the other. If the shorter recognition helix is longer than m residues (m was set to eight), “overhangs” are allowed, such that some of the residues of the shorter recognition helix are not mapping to any residues in the longer recognition helix. The maximum amount of permissible overhang is such that at least m residues are overlapping between the two recognition helices. If both recognition helices are of the same length and are longer than m residues, then the above considerations apply as well (which helix is treated as shorter is irrelevant). A final issue is that one recognition helix may run in the N-terminus to C-terminus direction, while the other runs in the opposite direction; this has to be taken into account as well. Figure 10 depicts all the possible mappings for a given pair of recognition helices. For each DNA-based alignment of a pair of HTH-DNA complexes, this procedure is carried out, and the recognition helix-based alignment with the lowest RMSD returned. Finally, out of all possible 50 alignments, the one yielding the lowest RMSD is returned as the final alignment. This pairwise alignment includes a residue-by-residue correspondence between the recognition helices of the two HTH-DNA complexes, as well as a base-by-base correspondence between their DNA molecules.

#### Clustering

The alignment procedure described so far yields a pairwise alignment between pairs of HTH-DNA complexes. We sought a multiple alignment that would yield a unified coordinate system across the database, where a DNA base (or amino acid residue) in one HTH-DNA complex would map to corresponding DNA bases (or amino acid residues) in all other HTH-DNA complexes. To obtain such a multiple alignment and its resulting unified coordinate system, the Affinity Propagation (AP) [79] clustering algorithm was run on the complexes in the database, with the distance between any two complexes defined as the final RMSD value of the alignment obtained from the pairwise structural alignment step. The AP algorithm has the advantage of returning an exemplar for every cluster found. Exemplars are characterized by being the cluster member with the smallest distance to all other members of the cluster. Furthermore, the AP algorithm does not require an explicit specification of the number of clusters to be returned, but instead uses a soft parameter approach that enables biasing toward smaller or larger clusters. By varying this single soft parameter and rerunning the AP algorithm, a clustering configuration was found that yielded a single, large cluster, which included the majority of HTH-DNA complexes, and a set of smaller clusters, mostly comprising one HTH-DNA complex each. Inspection of the singleton clusters revealed that they were either false positives that were not detected during the earlier stages of our pipeline, or protein-DNA complexes in which the DNA molecule was substantially bent. Because these complexes deviated markedly in structure from most HTH-DNA complexes and formed only a small subset (nine proteins), they were excluded from the analysis used in deriving a unified coordinate system. However they were retained in the database, as a separate set, to facilitate their future analysis. All false positives were removed entirely. Using the exemplar of the cluster as a reference point, the pairwise alignments between every HTH-DNA complex and the exemplar complex were used to establish a multiple alignment. A correspondence between any two complexes can be found by first mapping to the exemplar complex, and then mapping to the other complex. For example, if the ith DNA base of complex 1 mapped to the jth base of the exemplar, and the jth base of the exemplar mapped to the kth base of complex 2, then the ith base of complex 1 maps to the kth base of complex 2. Using this scheme, a single unified multiple alignment was determined. In addition, all HTH-DNA complexes other than the exemplar were affine transformed so that their DNA molecules and recognition helices are superimposed on the exemplar complex, to prepare the final database.

### Database schema

**Fig. 11:**
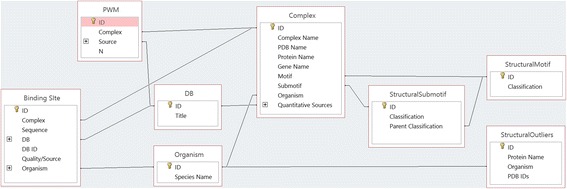
Database schema showing all major tables and their inter-relationships

The full schema of the database is shown in Fig. 11. The “Complex” table is the central table, whose entries correspond to the unique protein-DNA complexes in the database. Each complex corresponds to a single protein domain that binds DNA. This table contains information on the PDB id of the structure, gene and protein name information, classification of the motif, source organism, and a listing of database sources with quantitative binding information for the complex. Two other major tables, as well as a number of minor tables, support the “Complex” table. The “Binding Site” table contains entries corresponding to unique and experimentally verified DNA binding sites. Each entry identifies the complex and corresponding DNA binding sites, the source database, the quality and type of experiment used to identify the site, and the source organism. The “PWM” table contains information about the PWMs of each complex in the database, including IDs corresponding to the files containing the actual PWMs and a listing of all sources used deriving the PWM. In addition to the core database which contains all the meta information, formatted files containing PWMs and processed PDB files containing the full complexes, the HTH domain in complex with the DNA, the HTH domain alone, and the DNA molecule alone are also available for download from the website.

## Utility and discussion

**Fig. 12:**
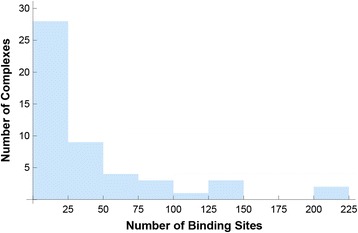
Distribution of binding sites. A histogram of the number of experimentally-characterized DNA binding sites found per protein-DNA complex is shown

**Fig. 13:**
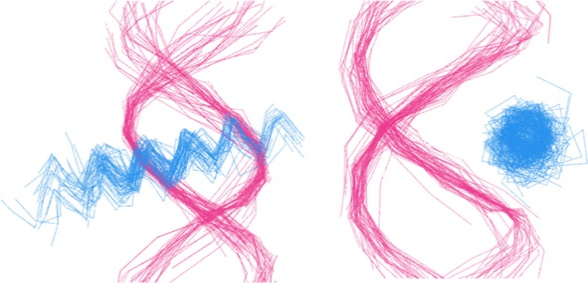
Structural alignment of HTH-DNA complexes. Two views (different viewing angles) of the 63 structurally aligned HTH-DNA complexes in the final database. The C_α_ traces of recognition α-helices are shown in blue and the C_3’_ traces of DNA helices are shown in pink

**Fig. 14:**
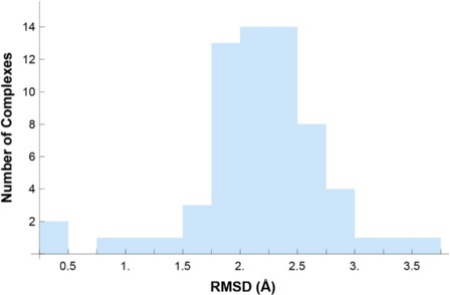
Structural similarity of HTH-DNA complexes. A histogram of the distribution of RMSD values for the HTH-DNA complexes in the database is shown. RMSD values are computed based on the distance of each complex to the exemplar

We obtained 63 non-redundant HTH-DNA complexes and PWMs. The complexes are listed in Table 3, and the data sources used in deriving the PWMs are listed in Table 4. Over 2,100 DNA binding sites were found, spanning over 60 distinct HTH domains and 30 organisms (Table 5). The number of DNA binding sites found per protein-DNA complex ranged from 1 to 210, with a median of 19 (Fig. 12). The structural alignment and clustering steps we performed resulted in a single cluster that included all the HTH-DNA complexes in the database. Figure 13 depicts the aligned complexes, and Fig. 14 shows the distribution of RMSD values from all cluster members to the exemplar structure (closest structure to all others). The RMSD values are low, with a median of around 2 Å and no values above 3.5 Å, indicating that the alignments are within experimental accuracy. This is confirmed by the tight visual superposition (Fig. 13) indicating that the structural alignment was successful and that the HTHs selected for the database do bind with a highly conserved binding modality that can be exploited algorithmically using our unified coordinate system.

**Table 3:** List of final set of structures in database. Some PDB files contain multiple non-redundant HTH domains which were treated as separate structures

ID	PDB ID	Chain ID	Recognition helix residues	ID	PDB ID	Chain ID	Recognition helix residues
1	1AWC	A	371–383	33	1 K61	A	172–189
2	1 AU7	A	44–51	34	1 K78	A	132–140
3	1 AU7	A	142–157	35	1 K78	A	62–75
4	1B72	A	244–262	36	1 K78	B	386–396
5	1B8I	A	142–157	37	1L3L	A	201–217
6	1B8I	B	245–260	38	1LE8	A	110–124
7	1BC8	C	55–70	39	1LE8	B	172–188
8	1BL0	A	41–52	40	1LMB	3	44–51
9	1BL0	A	91–102	41	1LQ1	A	208–226
10	1CF7	A	55–68	42	1O3S	A	179–193
11	1CF7	B	113–132	43	1PDN	C	47–60
12	1D5Y	A	34–47	44	1PER	L	28–36
13	1DDN	A	38–50	45	1PP7	U	79–90
14	1DU0	A	41–57	46	1PUE	E	227–240
15	1DUX	C	56–68	47	1PUF	A	245–268
16	1E3O	C	43–53	48	1PUF	B	276–294
17	1E3O	C	141–157	49	1R71	A	181–190
18	1EFA	A	16–25	50	1RIO	H	408–424
19	1F4K	A	53–67	51	1RZR	A	15–24
20	1FJL	A	42–63	52	1SAX	A	41–55
21	1FOK	A	104–116	53	1TC3	C	236–244
22	1GDT	A	172–180	54	1U78	A	92–103
23	1GXP	A	192–206	55	1U8R	A	37–51
24	1HCR	A	172–180	56	2CGP	A	180–192
25	1HLV	A	119–130	57	2HDD	A	42–57
26	1HLV	A	38–48	58	3CRO	L	28–36
27	1IC8	A	140–150	59	3HDD	A	42–57
28	1IC8	A	260–273	60	6CRO	A	27–36
29	1IG7	A	141–159	61	6PAX	A	117–130
30	1IGN	A	538–552	62	6PAX	A	47–60
31	1JE8	A	183–198	63	9ANT	A	42–58
32	1JGG	A	141–159

**Table 4:** Distribution of DNA binding site data sources

Data source	Fraction of DNA binding sites
TRANSFAC	39.39 %
PRODORIC	22.41 %
RedFly/FlyReg	18.22 %
Fly SELEX	17.79 %
Original literature	14.13 %
JASPAR	12.04 %
HTP SELEX	4.19 %
RegTransBase	2.90 %
DBTBS	2.19 %
RegulonDB	1.05 %
DPInteract	0.71 %

Percentages do not add up to 100 because some sites occur in multiple databases

**Table 5:** Distribution of source organisms for DNA binding sites

Data source	Fraction of DNA binding sites
*Homo sapiens*	36.39 %
*Drosophila melanogaster*	23.74 %
*Mus musculus*	22.17 %
*Escherichia coli*	19.65 %
*Rattus norvegicus*	12.84 %
*Xenopus laevis*	11.13 %
*Hylobates lar*	8.28 %
*Gallus gallus*	8.28 %
Cricetulus griseus	8.28 %
*Cercopithecus aethiops*	8.28 %
*Bacillus subtilis*	7.99 %
*Tribolium castaneum*	4.19 %
*Drosophila pseudoobscura*	4.19 %
*Drosophila funebris*	4.19 %
Others	3.62 %
*Sus*	2.85 %
*Saccharomyces cerevisiae*	2.00 %

Percentages do not add up to 100 because some sites occur in multiple organisms

### Core functionality

The database is available in downloadable form for programmatic use, and as a web service for interactive use. Users are able to browse and search for HTH-DNA complexes using all available fields, including gene and protein names, motif types, and source organism. For each entry, graphical and numerical representations of the PWM are readily accessible on the website, in addition to information describing the mapping of the structure to the unified coordinate system.

### Protein-specific statistics of the HTH-DNA binding interface

**Fig. 15:**
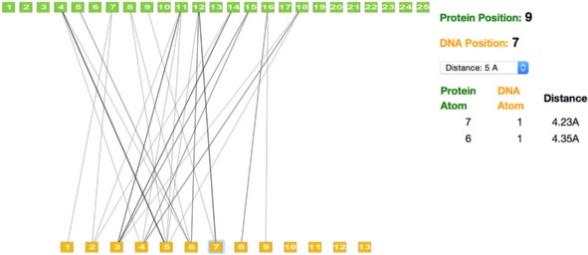
Snapshot of web-based interface for analyzing atomic contact frequencies of HTH-DNA interactions. The protein-DNA binding interface of the Ultrabithorax Homeodomain from *D. melanogaster*. Green and yellow squares represent residue positions of protein and DNA, respectively, which have been mapped to the universal coordinate system. Edges connecting squares indicate proximity of atoms within a user-defined threshold (shown at five angstroms). For each position pair, the user can interrogate the set of atom-atom interactions observed

For each HTH-DNA complex, the statistics of pairwise atomic contacts are visualizable on the website (Fig. 15). After the user selects a distance cutoff, a high-level summary of the most important residue position pairs is presented, with thickness of edges between positions indicating frequency of atomic contacts. This enables the exploration and identification of potentially specificity-determining residues in different HTH proteins. Furthermore, the user is able to select specific residues for further analysis. The interactive website provides a listing of all observed atomic contact pairs in the database for the selected positions, including their separation distance and atomic types.

### Global statistics on HTH-DNA interactions

**Fig. 16:**
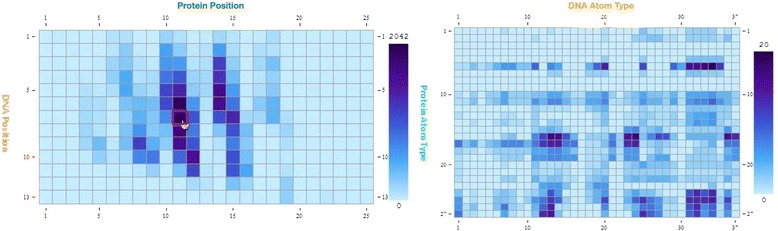
Snapshot of web-based interface for analyzing global properties of HTH-DNA interactions. Shown is a Heatmap of atomic contact frequencies averaged across all structures in the database. For any given residue position pair, one in the protein and one in the DNA (*left, highlighted in red*), users can inspect atomic contact frequencies across all atom types at a user-defined separation distance (*right*)

The web service also provides summary information on HTH-DNA interactions across all complexes. A “global” interactive widget is provided in which the user can observe the overall frequencies of atomic contacts across all position pairs in the unified coordinated system. Visual inspection immediately identifies positions 10, 11, 14, and 15 in the protein interface as the most important (Fig. 16), and similarly positions 5 through 10 in the DNA molecules. Consistent with biophysical intuition, it is readily evident using this widget that most interactions occur in the middle of the DNA sequence motif, with a gradual falling off of interaction frequency as one moves toward the periphery of the DNA motif. For more in depth investigation, the user is able to select any position pair of interest, as well as an interaction distance cutoff. Detailed information is then presented on the atomic interaction frequencies across all atom types in the form of a Heatmap (Fig. 16). As expected, atoms from residues known to be involved in mediating protein-DNA interactions, for example arginines and lysines, are significantly overrepresented in this interaction Heatmap (atom types 17 and 18 in Fig. 16). More broadly, this widget enables answering a wide array of questions, such as (i) when a given DNA position is a certain nucleotide, what are the most frequent protein atoms and residues interacting with it, (ii) conversely when a given protein position is a certain amino acid, what are the most frequent DNA atoms and nucleotides interacting with it, (iii) what are the relative interaction frequencies of different types of atoms, for example those in the backbone versus those in the side-chains. In general, this functionality enables the investigation of the biophysical determinants of protein-DNA selectivity in HTH domains.

### Development of sequence-structure algorithms

In addition to interactive use, the major utility of this database is to provide numeric access to the statistics of HTH-DNA interactions using a unified coordinate system that links structural and sequence information. Without this mapping, it is not possible to use supervised machine learning methods that use structural information as input and PWM information as output. We previously used this database in this precise fashion to derive de novo and statistical protein-DNA potentials that rely on combining structural and sequence data [26, 27]. These algorithms improved protein-DNA prediction performance beyond existing algorithms, and this improvement was shown to be due in part to the integration of structural and sequence information [27].

## Conclusions

The database described in this work will facilitate a number of unique applications. First, the coupling of structural information with binding affinity data enables the statistical analysis of the structural basis of protein-DNA biochemical affinity. Second, the unified coordinate system enables a comparison of the similarities and differences of the steric and physico-chemical environments at the interface of HTH-DNA binding at single-residue resolution. Third, the standardization of all complexes in the databases facilitates machine learning and data-driven applications that require structured and standardized data sets. Taken together these features will enable the exploration of sequence- and structure-based approaches to protein-DNA modeling.

## Availability and requirements

The service and database is freely available for academic use at http://ProteinDNA.hms.harvard.edu.

## Abbreviations

AP: Affinity propagation
ChIP: Chromatin immunoprecipitation
dsDNA: double-stranded DNA
HTH: Helix-turn-helix
ICP: Iterative closest points
NMR: Nuclear magnetic resonance
PDB: Protein data bank
PWM: Position weight matrix
RMSD: Root mean square deviation
SELEX: Systematic evolution of ligands by exponential enrichment
